# Understanding Health Promotion Policy Processes: A Study of the Government Adoption of the Achievement Program in Victoria, Australia

**DOI:** 10.3390/ijerph15112393

**Published:** 2018-10-29

**Authors:** Brydie Clarke, Boyd Swinburn, Gary Sacks

**Affiliations:** 1 Global Obesity Centre, Centre for Population Health Research, Deakin University, Burwood 3220, Australia; boyd.swinburn@auckland.ac.nz (B.S.); gary.sacks@deakin.edu.au (G.S.); 2 Prevention and Population Health Branch, Department of Health and Human Services, Melbourne 3000, Australia; 3 School of Population Health, University of Auckland, Auckland 1010, New Zealand

**Keywords:** obesity prevention, policy processes, health promotion, systems thinking, health policy, advocacy

## Abstract

Despite the growing health and economic burden associated with overweight and obesity, preventive policy progress has been deficient globally. This study investigated the policy process involved in the adoption of the Achievement Program, a settings-based health promotion intervention that was a key pillar of the Healthy Together Victoria obesity prevention initiative. The qualitative study utilised multiple theories of the policy process, as well as Causal Loop Diagramming (CLD) methods, to understand the policy systems underlying the decision to adopt the Achievement Program. Factors that impacted this obesity prevention policy adoption included problem prioritisation at Federal and state government levels; political risks regarding policy action and inaction, and framing used by policy advocates to reduce risks and highlight the opportunities related to the Achievement Program policy implementation. The use of CLD methods was advantageous to further conceptualise potential leverage points and effective ways to influence obesity prevention policy in future. As such, the findings contribute to the obesity prevention policy evidence base and toward developing a number of recommended actions for policy actors seeking to increase future policy action.

## 1. Introduction 

Obesity has become a global health challenge due to its rising prevalence and the associated burden of disease [1]. Elevated body mass index (BMI) accounts for approximately 2.8 million deaths each year [2], through increased risk of cardiovascular diseases, diabetes, cancers, chronic respiratory diseases and many other associated conditions [2,3]. Overweight and obesity is of particular concern for children and young adults given that obesity ‘tracks’ strongly into adulthood [4]. Consequently, interventions targeting children and young adults have been identified as crucial for obesity prevention [5]. Experts have identified the need for comprehensive settings- and community-based responses to enable children and adults to maintain a healthy body weight [3,6]. The World Health Organization’s (WHO) Health Promoting Schools (HPS) Framework is one example of a settings-based approach to support obesity prevention among children [7]. The HPS framework seeks to integrate health education into the curriculum, promote changes to school physical and social environments and engage families to create communities more supportive of healthy behaviours [7]. The HPS approach has been increasingly applied in various contexts and has been found to be an effective response to growing obesity prevalence [8,9].

In 2011, the Victorian State Government, Australia, adopted the Achievement Program initiative, based on an extended version of the HPS structure [10]. The Achievement Program encompassed a quality framework that could assist a broad range of settings to create healthier environments across a number of priority areas. These priority areas included healthy eating and physical activity, mental health and alcohol misuse, amongst others. AUD 4 million-implementation funding for Achievement Program was primarily used to fund central co-ordination to help support schools, early year’s settings (e.g., kindergartens) and workplaces to create environments conducive to healthier behaviours. The Achievement Program was proposed and was eventually adopted as a voluntary policy, with participating organisations required to meet set benchmarks in order to be recognised as health promoting [10]. The Achievement Program initiative formed part of a broader systems-based initiative designed to prevent obesity in Victoria, known as Healthy Together Victoria (HTV) that was active from 2011 to 2016.

In recent years, there has been emerging research investigating obesity prevention policy processes to understand facilitators of and barriers to progress in policy adoption [11]. Previous research has identified some of the policy process determinants that influence obesity prevention policy decision-making, including individual skills, knowledge and capabilities of policy actors, and the processes within political institutions that shape policy adoption [12,13]. Other influences on policy decisions include the power dynamics of networks and groups involved in policy development, as well as socio-political and economic factors that shape individual policy maker’s ideas and beliefs [14,15]. However, most of the existing literature is ‘a-theoretical’ in nature [11], and consequently, provides an incomplete understanding of obesity prevention policy processes [16]. Where studies have employed ‘theories of the policy process’, substantial methodological weaknesses have remained [11]. Furthermore, the existing literature has also highlighted how each individual theory may be limited in explanatory power [11]. Hence there is the need for studies utilising multiple comprehensive theoretical approaches in order for advocates to best understand how to intervene and enable policy change. 

This study sought to contribute to the obesity prevention policy evidence base by investigating the processes involved in the adoption of the Achievement Program health promotion intervention by the Victorian Government, using multiple theories of the policy process [17]. In doing so, the study sought to answer the questions of how and why [16,18], this initiative was adopted by Victorian policy makers. The aim was to help understand potential ways to drive future obesity prevention policy change in this context. To help bring together the theoretical analysis, the research also sought to utilise Causal Loop Diagramming techniques which can provide a heuristic tool for understandings complex policy systems [19]. 

## 2. Materials and Methods 

### 2.1. Research Scope 

This study focused on the Victorian state government policy formulation and decision-making processes related to the initial decision to fund the Achievement Program initiative as part of HTV. The study did not consider factors influencing policy implementation. It is recognised however, that policy processes did not necessarily occur in a sequential manner [20,21,22] and therefore some information relating to Achievement Program implementation and evaluation planning was considered within this analysis where relevant. Subsequent decisions pertaining to the refunding of the initiative were not examined here. The overall policy process studied was from 2008 to the end of 2012. 

### 2.2. Research Setting

This study was undertaken whilst BC was based within the Victorian State Government Department of Health and Human Services (DHHS) whilst undertaking her PhD research. The student placement enabled the research to be developed and conducted within the ‘real-world’ policy context, and was designed to allow a thorough understanding of the political environment, and to facilitate data collection. The location of the researcher within the policy context also influenced the epistemology of the study, with an interpretivist theoretical perspective used throughout. This lens acknowledges that prior understandings and prejudices shape the interpretation and subsequent data collection and analysis [23].

### 2.3. Data Collection

#### 2.3.1. Semi-Structured Interviews 

A total of 10 interviews were conducted with participants identified through snowball, purposive sampling. Interviewees included politicians, political advisors, civil servants, academics and other stakeholders (e.g., relevant NGO representatives). All but one invited participant took part in the study. Initial interviewee identification was facilitated through the researcher’s location within the policy context. 

As per the ethics requirements, participants could elect to maintain their organisation’s confidentiality to reduce any risk of identification. Hence, when participant quotes are provided in this paper, their organisations are not provided. 

Interviews took place between December 2015 and October 2016. The duration of interviews ranged from 16 min to 75 min. All interviews were audio recorded and participants were given the opportunity to review transcripts for accuracy. A semi-structured interview guide was used for the interviews, and was based on a systematic review of the literature [11] and the political science frameworks underpinning the study (see Supplementary File S1). 

#### 2.3.2. Documents 

Interview participants were asked whether they could suggest any documentation that may be relevant to the investigation. Documents that detailed information relating to the policy decision-making were collected in an iterative manner, concurrent to the conduct of interviews. Documents were excluded if they did not provide insight into the Achievement Program policy decision-making processes. For example, several documents focusing on very detailed aspects of policy implementation were excluded from analysis. Documents that were retained within the dataset included formal studies and evaluation reports, meeting agendas and minutes and other written reports of events, administrative documents such as government briefings, Hansard transcripts, letters, memoranda, and PowerPoint slides. As the policy decision-making process occurred largely within the government setting, there were no relevant media and stakeholder submission documents for inclusion. A total of 17 relevant documents were included for analysis.

Documents that were published and therefore publicly available were referenced accordingly. Where internal DHHS documents were used, these were referred to as DHHS policy documentation, with the relevant year listed. 

### 2.4. Data Analysis 

Deductive thematic analysis was conducted using the conceptual and relational themes articulated through the selected theories of the policy process [24,25]. The selection of the theories was informed by the aforementioned systematic review [11] which highlighted the value of synthesis theories of the policy process that provide particular utility to explain both policy stasis *and* policy change [26]. These are the Advocacy Coalition Framework (ACF) and Multiple Streams Theory (MST). 

The ACF focuses on how public policy alterations are driven by coalitions of individual policy actors [27]. The theory assumes that policy actors, which include legislators, civil servants, journalists, academics, and members of not for profit organisations, are ‘boundedly rational’, and due to limited cognitive abilities, they simplify the world through their existing belief systems [28]. Accordingly, policy actors potentially bias the interpretation of information, evidence and experience during policy processes [27]. The ACF suggests that coalitions form based on shared beliefs, and aim to disseminate information and influence policy in line with their key tenets [21]. 

The ACF considers the role of policy systems, broader societal contexts and external events (e.g., changes to public opinion or crises) that shape opportunities for coalitions to impact policy decision-making processes [26]. Relatively stable parameters such as the basic constitutional structure are also important influences within the ACF [26]. The framework recognises that not all policy change is radical and can instead occur as a result of policy learning [29], whereby the beliefs of policy actors are altered with experience and/or access to new information [26,30] or through negotiated agreements facilitated by ‘policy brokers’ [31].

The ACF has previously been described as being insufficiently capable of explaining institutional factors affecting policy change [27,32,33,34,35]. In order to address this critique and strengthen the ACF analysis, constructs from the Institutional Analysis and Development Framework (IADF) were integrated as part of the ACF analysis. The IADF notes how institutions are “…shared concepts used by humans in repetitive situations organised by rules, norms, and strategies” ([36], p. 23). The framework also focuses on policy ‘action arenas’ that are shaped by broader organisational norms and rules which can be crucial influences on policy decision-making [37]. Hence, the IADF places less emphasis on how individual rationality or beliefs shape policy processes [38,39]. 

The MST suggests that policy formation is the result of three separate streams aligning: problems, policies and politics [40]. The problem stream relates to issues that require government action [41], which is affected by factors such as the availability of indicators and information, how issues are framed, current government conditions (e.g., budget deficit) and focusing events (e.g., crises or changes of government). The policy stream focuses on the solutions available to address identified problems, which take time to develop and evolve as numerous policy actors shape them [42]. The third, political, stream refers to the effect of the broader political discourse, which is influenced by ‘national mood’, pressure group advocacy, and administrative or legislative turnover [40]. The MST also identifies ‘policy entrepreneurs’ (individuals, coalitions, power brokers and organisations [42,43]) that invest time, energy, reputation and money towards policy change [26]. These policy actors, ‘couple’ the three streams when ‘policy windows’ of opportunity arise [40], through external influences such as budgetary crises [44] or natural disaster [45]. 

#### Causal Loop Diagrams (CLD)

In order to gain a sense of how the influencing factors relate to each other, a CLD of the Achievement Program policy process was developed using the approach outlined by Kim and Anderson [46]. This involved analysing the theoretical analysis text data for either explicitly described or implied causal linkages between policy influences. For the purposes of developing labels for the CLD diagram, each fragment of text was then translated to a ‘microstructure’, defining both cause and effect variables. These data excerpts, their associated microstructures, as well as the direction and polarity of connections between variables were recorded in coding tables (see File S2). NVivo™ software (QSR International, Melbourne, Australia) supported the first stage of this analysis, and the documentation of associations between factors was undertaken using Vensim™ software (QSR International, Melbourne, Australia) [47]. The variable names, direction of the linkages and association direction (i.e., polarity) were developed in an iterative manner [48,49]. 

### 2.5. Data Reporting

The findings bring together multiple sources and forms of data, and multiple theoretical lenses. Consequently, in order to simplify the reporting of results, an approach was taken whereby the learnings generated, and the supporting evidence (e.g., quotes, document excerpts) to substantiate findings, were presented when triangulation was achieved across data sources and methods (i.e., where various sources of both document sources and interviews support the learnings). Where learnings could not be triangulated, for example, if only interviews, or only one pertinent interviewee supported the findings generated, data excerpts note attribution to only a single data source. 

### 2.6. Ethics Approval

Ethical approval for the study was granted by the Deakin University Human Research Ethics Committee 2015 (HEAG-H 106_2015). Approval for the conduct of the research was also granted by the DHHS in 2015.

## 3. Results 

**Figure 1:**
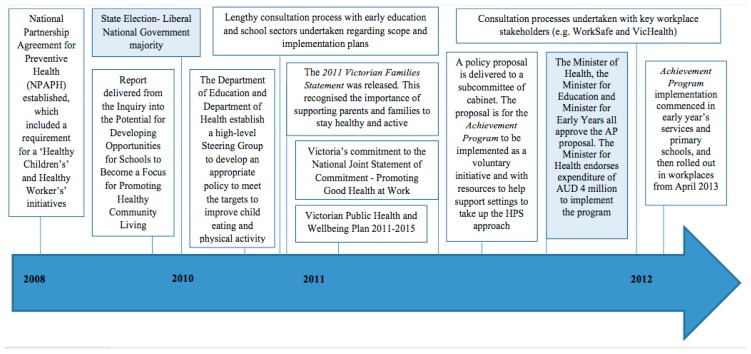
Policy processes involved in the adoption of the Achievement Program overview.

The key processes undertaken and milestones related to the adoption of the Achievement Program by the Victorian government are summarised in Figure 1. In November 2011, the Liberal Party Australia (LPA) party defeated the Australia Liberal Party (ALP) party in the Victorian state election. An indirect consequence of this was the defunding of the Kids Go For Your Life (KGFYL) obesity prevention initiative that had been in place for several years. At the same time, planning was underway for Victoria’s implementation approach to the Australian National Partnership Agreement for Preventive Health (NPAPH) which required the delivery of both ‘Healthy Children’s’ and ‘Healthy Worker’s’ initiatives. The NPAPH involved investment of AUD 642 million, over six years, to tackle chronic diseases caused by obesity (healthy eating and physical activity being the key drivers of focus), as well as tobacco and alcohol misuse [50]. Consequently, the DHHS, in collaboration with the Department of Education and Training (DET) developed the Achievement Program policy proposal. This proposal was also developed providing consideration to the recommendations made from an *Inquiry into the Potential for Developing Opportunities for Schools to Become a Focus for Promoting Healthy Community Living* held in 2010. The Achievement Program proposal was reviewed and endorsed by a subcommittee of cabinet, before the final decision to adopt the *Achievement Program* (both Children’s and Workplaces) was made by the Minister of Health, on 5 August 2011. 

### 3.1. Advocacy Coalition Framework Analysis 

#### 3.1.1. External Subsystem Events 

The ACF notes that external events such as changes in public opinion or socio-economic conditions can trigger policy change [28]. The Achievement Program policy adoption was facilitated by the Federal government’s NPAPH in 2008. This agreement meant that there was both a mandate for action and resources committed for obesity prevention initiatives in Victoria [51].

*“It [the emphasis of the need for the policy] was all around the National Partnership Agreement and we had a mandate and money to do something…and we had targets [that] we had to meet from the Commonwealth so there was an imperative for us to do something.”*

*(Senior Policy Maker 1)*

A Victorian Parliamentary *Inquiry into the Potential for Developing Opportunities for Schools to Become a Focus for Promoting Healthy Community Living* was another significant external event. This inquiry provided clear recommendations for a health promoting schools approach to be implemented in Victoria as well as a “…formal recognition and award program to acknowledge and celebrate outstanding achievements by schools, communities and individuals in promoting healthy community living” ([52], p. xii). 

A change in elected government, in 2010 was also a noteworthy event. This change resulted in the cessation of the ‘Kids Go For Your Life” (KGFYL) health promotion initiative. Whilst there was no evidence that public backlash occurred in response to the termination of the KGFYL program, the possibility of this was identified as a potential political risk by policy makers given there may be a perceived ‘service gap’ in the area of child health promotion. Policy makers therefore saw the Achievement Program as a way to meet this ‘gap’.

*“There were some incredibly tight timeframes around getting something up and running because KGFYL was ceasing so there was, from a department and ministerial perspective, there was a risk in having any great length of delay in going from the KGFYL”*

*(Policy Maker 1)*

The Victorian Government’s commitment to improving workers’ health through the Victorian Public Health and Wellbeing Plan 2011–2015 and the Australian Government’s *Joint Statement of Commitment* on worker health facilitated the inclusion of workplace settings within the Achievement [53]. In addition, the WorkSafe Healthy Workplace Check initiative, that was in place at the time of the Achievement Program policy development, was undergoing a review and was potentially being discontinued [54]. This presented government with a risk pertaining to a policy gap in relation to government workplace health and wellbeing initiatives. 

#### 3.1.2. Coalition Opportunity Structures 

The ACF notes that the ability for coalitions to progress policy is influenced by long-term opportunity structures, such as the degree of consensus required for change and the openness of the political systems [31]. As a health promotion program (rather than legislative or fiscal policy), the Achievement Program policy was not required to pass through cabinet. Policy makers did elect to engage in considerable consultation with stakeholders and, to some degree, the public. Interview participants explained that the focus of consultations was to increase support from key stakeholders who would be critical to the successful implementation of the policy (e.g., schools, workplace peak bodies).

*“…What we foresaw before we engaged [a stakeholder organisation] was that they could see the Achievement Program as competition and we didn’t want that to be the case…we wanted to engage them right from the beginning to talk about how we weren’t trying to be in competition with what they were doing”*

*(Policy Maker 4)*

However, the broad consultations resulted in a larger policy system of actors involved in the Achievement Program policy processes and increased the time required and complexity of policy change. 

### 3.2. Action Arenas and Patterns of Interaction (Institutional Analysis and Development Framework) 

The IADF notes how policy decision-making ‘action arenas’ are shaped by broader organisational norms and rules that are reflective of the external community context [37]. The children’s Achievement Program policy arena involved collaboration across both the DHHS and DET. DHHS policy brokers strategically set up the children’s Achievement Program governance structure so that there was shared decision-making powers across both DET and DHHS, which was an identified enabler for the policy approval.

*“So the network and the distribution of power; that original steering group really only had two representatives from the Department of Health and about eight from Department of Education, so we deliberately sought to shift some of that power to Education as a strategy to engage them, to give them a little bit more power and control over the direction to inform it right from the beginning.”*

*(Policy Maker 2)*

Interviewees reflected that this across department governance model was not necessarily reflective of the usual government arrangements for development of such a policy. 

Policy brokers were also strategic in seeking approval for the Achievement Program policy from an existing DET/DHHS Interdepartmental Committee (IDC), as well as from another existing body (the Children Services Coordination Board) that served to enable a whole-of-government policy approach to improve child outcomes [55]. The requirement to obtain these additional approvals meant further inspection of the proposed Achievement Program policy, which delayed the overall process. However, policy brokers provided evidence of these comprehensive reviews when presenting the Achievement Program proposal to the final decision-makers (i.e., the respective Ministers). This helped to demonstrate the collective support and confidence in the Achievement Program approach, and was therefore identified as a facilitator for the policy adoption. 

In contrast, the workplace’s Achievement Program initiative was developed by the DHHS, in consultation with a number of peak bodies (e.g., the Victorian Employers Chamber of Commerce and Industry, WorkSafe), without any formal partnerships across government. Therefore, the policy did not receive the same degree of examination as the children’s Achievement Program across government departments and was endorsed through a more streamlined approval process. 

### 3.3. Institutional Rules and Procedures

The IADF was useful for examining institutional rules and how these shaped the ability of policy actors to influence decisions. Institutional rules guided the allowable set of behaviours of policy participants within the Achievement Program policy processes. For example, decision-makers were required to provide details regarding whether the Achievement Program could result in an increased regulatory burden on businesses, when presenting to the sub-committee of Cabinet. By crafting the proposed Achievement Program as a voluntary program, policy makers circumvented the need to justify an increased regulatory burden that would have resulted from a mandatory program. The voluntary nature of the policy was therefore perceived as a facilitator for policy adoption.

The proposal to the sub-committee of Cabinet also required information regarding whether there was an evaluation strategy developed to enable the measurement of ongoing effectiveness of the policy. The body of evidence of existing HPS frameworks provided useful input to meet this institutional expectation to facilitate approval of the policy. 

#### 3.3.1. Advocacy Coalitions 

The ACF assumes that the participants within policy subsystems form coalitions based on agreement on ‘core’ and/or ‘policy beliefs’ [28]. Whilst there were proponents working together to advance the Achievement Program policy, this analysis did not find evidence of a coordinated coalition opposing either the workers or children’s components of the Achievement Program. There were however, a small number of opponents who were primarily located within organisations that would be tasked with the implementation of the Achievement Program policy. These opponents were resistant to the policy primarily due to the resources that would be required to implement the framework. In addition, a number of opponents demonstrated some contention in the value of the Achievement Program above existing initiatives. 

#### 3.3.2. Belief Systems 

The ACFs notion of belief structures was an evident influence in the Achievement Program policy decision-making. Specifically, the core beliefs (i.e., very broad and largely immovable beliefs, such as neoliberalism) and the policy beliefs (i.e., more specific beliefs regarding certain policy areas that are quite resistant to change) [28,56] were key influences on decision-makers.

### 3.4. Core Beliefs 

The ACF suggests that core beliefs, which are very broad and largely immovable beliefs, such as authoritarianism drive coalition behaviour [28,56]. Core beliefs regarding individual responsibility for obesity prevention that were held within the community and among stakeholders, were identified as a barrier to the government adopting the settings-based Achievement Program. As a result, the policy proposal was crafted as voluntary, as this was deemed more likely to get adopted.

*“So certainly from Worksafe’s point of view, something that came through very strongly was that anything in this [policy] space could only be voluntary.”*

*(Policy Maker 1)*

Interviewees noted how they had to “…tread carefully, in terms of [their] approach, how [they] positioned and communicated [the Achievement Program proposal] to workplaces” (Senior Policy Officer 3). 

### 3.5. Policy Beliefs 

According to the ACF, policy beliefs relate to what are considered the most appropriate policy response for identified problems [27]. Some policy makers believed that the Achievement Program would provide an effective and sustained obesity prevention policy response in a context where previous investments in obesity prevention had been intermittent. This provided substantial motivation for policy brokers to commit significant time and effort to progress the Achievement Program policy initiative. Many interviewees also reflected that some senior decision-makers (both bureaucrats and elected officials) held beliefs in support of environmental approaches to obesity prevention and that this was an important facilitator.

*“I do also think that the Secretary at the time understood that agenda—understood the prevention agenda and was also highly supportive of it.”*

*(Senior Policy Maker 2)*

However, a number of advocates recognised that the Achievement Program approach may contradict policy beliefs held by some decision-makers including Australian Liberal Party members who traditionally oppose policy responses of strong government intervention [57,58]. To counter this potential barrier, policy advocates demonstrated political astuteness by framing the Achievement Program policy as a central component required to deliver the Victorian approach to the NPAPH (which had been committed to by both state and federal governments).

*“…We were under a Liberal government, so we kept emphasising that the state has signed up to this, we have to do it, the money’s coming. [So that there was] no opportunity to shut this down, because we didn’t know what the Liberals would think. As it turned out we had a wonderful Minister who totally understood public health so it wasn’t really an issue but you just never know because sometimes the values don’t align.”*

*(Senior Policy Maker 1)*

Interviewees also provided evidence of ‘policy-oriented learning’, whereby evidence regarding the effectiveness of the WHO’s Health Promoting Framework policy was described as helpful in shifting policy beliefs in support of the Achievement Program. 

#### Policy Brokers 

There were a number of policy brokers who were identified as crucial in enabling the Achievement Program process and ultimately driving policy change. Interviewees suggested that these individuals used their positions within the policy decision-making systems to facilitate discussions with stakeholders and to successfully advocate to decision-makers that the Achievement Program should be endorsed.

*“[A Senior Public Health Advisor] did a lot of the heavy lifting with a lot of those individual groups, which was not always straightforward. There was a bit of push back, [but] they were, in the end, actually very supportive [of the Achievement Program].”*

*(Senior Policy Maker 2)*

### 3.6. Multiple Streams Theory Analysis 

#### 3.6.1. Problem Stream 

As described by Kingdon [40], problems can be elevated onto the agenda through indicators, information or ‘focusing events’, such as natural disasters or epidemics. The aforementioned lapsing of KGFYL could be considered a focusing event that helped to elevate the need for children’s health promotion efforts centred on improving dietary and physical activity behaviours. In addition, both the health and education sectors had identified the need for policy responses to support health and wellbeing in schools and early year’s services. Together, these factors increased the acceptability of the proposed Achievement Program policy with decision-makers. 

#### 3.6.2. Politics Stream 

There were a number of political factors that influenced the Achievement Program policy process. These included political party interests and consensus building among actors. 

### 3.7. Political Interests 

Interviewees reflected that the Achievement Program provided a vehicle to serve the political interests of the government of the day, and that this increased the acceptability of the proposed policy.

*“[The AP] delivered lots of good things for them [the Minister for Health], because there were lots of good news stories lots of photo ops [opportunities], lots of chances to go out and meet and greet.”*

*(Senior Policy Maker 1)*

With a change in Government occurring in 2010, the incoming Liberal party was also interested in implementing something ‘new’ that had not been delivered by the previous Labor government. This was seen as an enabler for the adoption of AP.

*“…The political cycle always has a role…so at that stage [with the change in government] you are usually looking for something new…we couldn’t have [KGFYL] anymore.”*

*(Senior Policy Maker 2)*

The political relationships across government were identified as facilitating the Achievement Program adoption, with the Minister for Health’s senior position within government seen as important for gaining across-cabinet support for the AP.

*“We were very fortunate with the factions within the government. When we were developing the AP, the Health Minister also had a good relationship with the Early Childhood Minister so there was a connection which helped.”*

*(Senior Policy Maker 1)*

#### Policy Stream 

The policy stream of the MST focuses on the solutions available to address identified problems, which evolve as numerous policy actors shape them over time [42]. Solutions that are technically feasible and framed to align with decision-maker objectives are more likely to be accepted by government [59]. Interviewees indicated that policy makers assessed evidence of effectiveness in relation to the HPS approach when developing the Achievement Program policy proposals. Interviewees also stated that implementation evidence (i.e., information regarding the implementation and viability of the health promoting schools approach adopted elsewhere) also helped to demonstrate the technical feasibility of policy implementation. The initiatives’ perceived relatively low cost, in comparison with other obesity prevention programs, was identified as an enabler by one senior decision-maker.

*“…It is not a high cost initiative and, given the reach, it was a reasonably easy thing I think for the government to agree to…”*

*(Policy Director 1)*

Participants identified that the internal capacity within the DHHS and DET was insufficient to oversee the implementation of the AP and therefore there would need to be an appropriate delivery health promotion organisation. However, interviewees reflected how the process of negotiating a delivery organisation also gave rise to conflict between public health organisations.

*“I would say the complication we had was working out the delivery, [with] the DHHS not being a delivery body. The decisions around, who do we fund to do this? I think that’s where some of the…well, kind of turf conflict [between public health organisations who seek funding to implement the health promotion programs] came out a little bit.”*

*(Policy Maker 1)*

### 3.8. Framing

In addition to altering policy frames to better align with decision-maker beliefs, as outlined earlier, there was evidence that ‘policy entrepreneurs’ [59] modified their framing of the Achievement Program to emphasise the potential rewards associated with its implementation. The potential contribution the Achievement Program could make toward broader government objectives was particularly highlighted.

*“[When one of the policy entrepreneurs] spoke to the Secretary about this approach, [they] would be talking about that the hospital system has quality measures, [and that] we are trying to do the same thing for prevention. So it was about creating the match between where [the Secretary] wanted to go and what this might deliver for [them].”*

*(Senior Policy Maker 2)*

The policy was also framed to align with other policy objectives including the aforementioned Parliamentary Inquiry, the NPAPH, the *2011 Victorian Families Statement* (which had recognised the importance of supporting parents and families to stay healthy and active), and the *Victorian Health Priorities Framework 2012–2022* (which included a specific action regarding the establishment of a new health promotion program). Government policy brokers were also deliberate in framing the Achievement Program as a key pillar that would enable the implementation of HTV. 

When consulting with stakeholders, policy advocates also used framing to minimise potential pushback from implementation organisations by highlighting how the initiative would sit alongside existing policies and not duplicate current efforts.

*“…It was about really articulating how the Achievement Program would help them [early childcare settings] meet the National Quality Standards that they had to meet and how it could support that for schools. It was about trying to line up the process and then demonstrate to schools how the quality improvement process that they went through with the Achievement Program was no different to the process that they were required to do otherwise by Department of Education. For workplaces, it was about demonstrating more the business case to them around the benefits, but these were all factors that enabled us to get the right kind of support to support its development.”*

*(Policy Maker 2)*

#### 3.8.1. Policy Windows and Entrepreneurs 

The MST notes how policy entrepreneurs must act swiftly when a policy window opens to get their proposals considered [26]. Through political capability, and dedicating time and effort, policy entrepreneurs were able to influence decision-makers to support the Achievement Program policy adoption.

*“…I give [one particular policy entrepreneur] an enormous credit for a lot because [they] really did drive it. [They] were a tough taskmaster but a pretty strong captain of the ship…that strong leadership [was] right up front.”*

*(Senior Policy Maker 2)*

#### 3.8.2. The Achievement Program Policy System 

**Figure 2:**
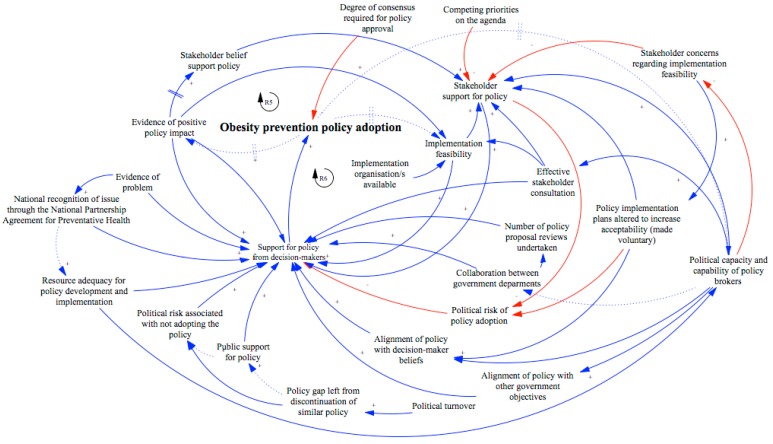
The causal loop diagram of influences on the decision to adopt the Achievement Program. All solid lines indicate where there was triangulated data (both across different methods (interview and documents) and source (different interviewees or document sources) to demonstrate the relationship between factors. Dotted lines were used to denote relationships whereby there was limited evidence within this dataset of a relationship and triangulation could not be achieved. Two hash marks, || on causal links between elements indicated a time delay between variables. A “+” sign and against the arrow head/handle and blue colour indicate that, as the causal variable increases or decreases, the influenced variable also changes in the same direction. A “-” sign and red colour arrow suggest the change is in the reverse direction.

Figure 2 consists of a CLD that depicts the interdependence of the policy determinants identified in this study, and reveals the dynamic and complex nature of policy decision-making in this context. 

This CLD highlights several central influences that were connected to numerous other policy influences, which indicates their potential power as a leverage point [60]. These influences included the political capacity and capability of policy brokers, stakeholder support for the policy, and the utilisation of a voluntary implementation approach. 

**Figure 3:**
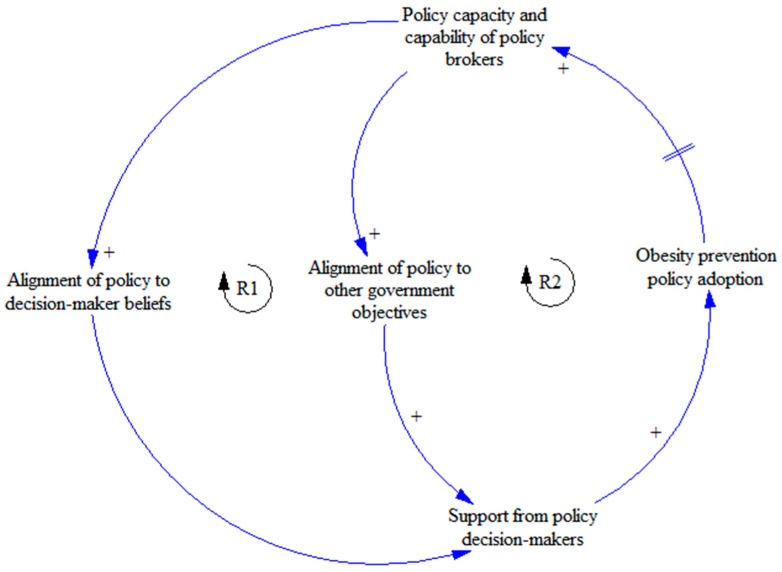
Achievement Program example leverage points for policy change. Reinforcing loops one (R1) and two (R2) demonstrate how improved political capacity and capabilities can increase (+) the alignment of policy proposals to decision-maker beliefs and other government objectives. This increased support from decision-makers for policy change. This becomes a virtuous reinforcing loop when reflection and learning regarding these effective tactics is shared with other public health policy brokers.

One example of a feedback structure that can increase the likelihood of obesity prevention policy adoption is increasing political capacity and capability of policy brokers to enable advocates to successfully reframe their arguments to better align with policy decision-makers’ beliefs and to broader government objectives. By better aligning proposals, decision-maker support for the Achievement Program was increased and consequently the policy change was able to occur. A virtuous reinforcing loop can be established, as indicated in Figure 3 (see Reinforcing loop 1 (R1) and R2), if professional experience in successfully securing policy adoption leads to an increase in the capability of policy brokers over time (e.g., through reflective practice). 

## 4. Discussion

This investigation of Achievement Program policy adoption processes, viewed through multiple theoretical lenses, demonstrated that there were multiple and interacting factors that influenced the Achievement Program policy adoption. Through the use of multiple theoretical frameworks, nuanced insights, as to how policy was altered as a result of various influences, were elucidated [18,61]. The findings identified how higher-level policies and a change in government created conducive conditions for the Achievement Program adoption. The analysis also highlights the tactics used by policy makers, such as framing the Achievement Program in a way that increased support from stakeholders and reduced the political risks associated with the policy. Utilisation of evidence regarding the general policy approach (i.e., the WHO’s HPO framework), as well as the learnings gathered from previously implemented policies also increased the perceived viability of the Achievement Program.

### 4.1. Stakeholder Resistance

The main barrier to the Achievement Program overcome by policy advocates was in relation to the resistance from implementation stakeholders in the education and workplaces sectors. This finding supports emerging studies underpinned by policy process theory that have highlighted the influence of stakeholder preferences on obesity prevention policy decision-making [62,63,64,65,66]. Policy brokers, who instigated a substantial period of consultation with stakeholders, were able to allay concerns and garner support for the Achievement Program policy. This process of engaging opposing factions has long been recognised in the political science literature as a key strategy to gain opposing group support on policy options [67,68]. However, to date, there has been little evidence of how this has occurred within the field of obesity prevention policy. Previous research has also uncovered that proposing policies as voluntary in nature assists to reduce the opposition to policy implementation from stakeholders and increase decision-maker receptivity [65]. However, the voluntary nature of the policy likely substantially reduces the reach of the intervention, which is likely to negatively impact policy effectiveness [40].

### 4.2. Beliefs

By using the ACF as an analytical lens, insights were developed regarding how policy makers’ core beliefs were influential in the decision-making processes, with the voluntary nature of the Achievement Program scheme being consistent with neoliberal ideology [65,69,70]. The study also demonstrated how decision-makers’ policy beliefs were facilitators of policy change, with the strong agreement regarding the appropriateness of the Achievement Program policy instrument recognised as an important enabler for policy action [70,71,72,73].

### 4.3. Framing

This study supports previous theoretically based evidence that uncovered how framing obesity prevention policies in a way that reduces the potential impacts of implementation helped subdue the opposition from stakeholders’ organisations [63]. Similarly, by framing the Achievement Program policy as a tool that would contribute to the achievement of broader state government policy objectives, stakeholder and policy maker receptivity to the initiative was increased. This is consistent with the findings of previous studies [65,73] that have noted the successfulness of this approach.

### 4.4. Institutional Factors

This study adds to the obesity prevention policy process literature through the detailed examination of institutional factors through the use of the IADF. Whilst previous studies of obesity prevention policy had identified interdepartmental collaboration broadly as potential facilitators [66,74], this study’s findings demonstrated how policy brokers were strategic in establishing organisational structures to support collaboration in order to increase policy receptivity with senior decision-makers. The analysis also highlighted how the Achievement Program complied with organisational rules (e.g., regarding the regulatory burden of proposed policies) that were in place at the time of the policy development and that this facilitated policy change.

### 4.5. Complexity of the Policy Process

Whilst recent years have seen the incorporation of complexity theory and complex systems methodologies into political science research [75], this study is one of the first to use CLDs to shed light on the interconnections in policy systems. In doing so, this research adds to existing studies of policy process by visually demonstrating the dynamics underlying obesity prevention policy decision-making. The CLD presented in this paper shares some similarities with the only other identified study to develop CLDs of policy systems, conducted in Fiji, which found that workforce capability and consultation with stakeholders were important drivers of policy change [76]. However, this previous study did not identify the potential negative impact that consultation may have in terms of increasing the time required for policy change.

The study findings are strengthened through several efforts to improve the credibility and dependability, including the use of verbatim quotes, provision of document excerpts and details regarding data analysis, collection and interpretation [77]. Triangulation using both methods (i.e., interviews and documents) and data sources (e.g., findings are consistent across different participants) also enhanced the dependability of findings [78]. Triangulation of interview findings with document sources was particularly important given the relatively small number of interviewees. The number of interviewees reflected the small number of individuals that were directly involved in the policy development process, with one potential interviewee inaccessible, as they had left the organisation. The inclusion of documents was also valuable given the potential for poor recall from participants given the time between policy processes and data collection [78,79]. The access to ‘insider’ policy participants and documents, achieved through the embedded research approach helped to ensure that pertinent data sources were included. The embedded study design also provided challenges for maintaining objective data collection, analysis and interpretation. A commitment to integrating a reflexive research approach was used to limit the potential for bias. Finally, it is acknowledged that the findings may have limited generalisability to other contexts. However, with the study applying theory to the analysis, the learnings may point to patterns and themes that can be applied elsewhere [17,80].

The study is also limited in that post adoption ‘policy cycle’ stages (e.g., policy implementation) were not considered. Hence, it remains unclear as to how certain strategies that secured policy adoption, such as the expedited consultation processes undertaken in the development of the workplaces Achievement Program, which helped secure policy adoption, may have impacted on the success of the Achievement Program implementation. Future research is therefore warranted to help to understand how characteristics of policy change processes impact on implementation effectiveness. Despite these limitations, the study findings can inform policy advocates, in similar contexts, in their efforts to influence obesity prevention policy-making. Specifically, the findings of this study indicate that policy advocates may find it useful to:

- Explicitly consider the policy implementation requirements (e.g., feasibility issues) at an early stage of the policy development process in order to identify potential risks of policy adoption and inform the development of risk management strategies (e.g., reframing policy solutions, consultations and negotiations strategies);
- Advocate the establishment of organisation structures that support collaboration and open, transparent policy development processes to assist in creating fairer opportunities for a broad range of external stakeholders to contribute to policy development. This is particularly important given the increasingly noted efforts of the food industry to influence policy makers, and the privileged access to decision makers that they often enjoy [81,82];
- Alter policy framing to align with broader government objectives, dominant belief systems and to minimise political risk (e.g., resistance from stakeholders).

## 5. Conclusions

This study investigated the policy process involved in the adoption of the Achievement Program as part of the HTV obesity prevention initiative. By utilising multiple theories, the study uncovered a number of key influences on obesity prevention policy decision-making, including the problem prioritisation at the Federal and then state government level; the political risks regarding policy action and inaction, and framing used by policy advocates to reduce risks and highlight the opportunities related to the Achievement Program policy implementation. This study found that both the MST and ACF were useful theoretical frameworks for unpacking policy processes, however the ACF analysis required strengthening through the incorporation of the institutional factors set out in the IADF. The use of CLD methods was advantageous to further conceptualise potential leverage points and effective ways to influence obesity prevention policy in future. Based on the findings of this study, recommended actions for policy actors seeking to increase future obesity prevention policy action in similar contexts include consideration of the policy implementation requirements early in the policy development process; identification of potential policy development risks, and development of corresponding risk management strategies (e.g., undertaking well-managed consultations and negotiations); and adapting policy framing to align with broader government objectives and dominant belief systems.

## Supplementary Materials

The following are available online at http://www.mdpi.com/1660-4601/15/11/2393/s1, File S1: Semi-structured interview guide, File S2: Example Qualitative Data Coding Chart Used to Develop Causal Loop Diagrams.
