# Supplementary material for: Understanding Health Promotion Policy Processes: A Study of the Government Adoption of the Achievement Program in Victoria, Australia
Source: Int J Environ Res Public Health. 2018 Oct 29;15(11):2393. doi: 10.3390/ijerph15112393 (PMC6265848; doi:10.3390/ijerph15112393)
Supplement: Supplementary file 1 [file ijerph-15-02393-s001.pdf]

Supplementary file 1: semi-structured interview guide

Warm up question:

1. My interest is the policy area of [insert]. Can you tell me a bit about your role in regard to this policy?

Key questions:

2. Why do you think the policy was [or was not] able to be adopted?
3. Now I'd like to talk about some of the broader contextual factors that influenced the decision-making related to the policy. Can you describe the key influences on the policy decision-making processes from your perspective?
   Prompts: Can you say more about that? Can you give me an example? Can you tell me a bit more about that?
4. Can you please reflect on any relevant broader political factors (e.g., free market versus government intervention) and how these influenced the policy, if at all?
5. How did timing influence in the policy process, if at all?
6. How was evidence used throughout the policy process, if at all?
7. I've observed many institutional factors that can influence policy (such as inter-departmental collaboration, role and involvement of other departments, role of things like treasury/budget reviews etc.) can you please comment on how these played a role in the adoption of this policy, if at all?
8. Can you please reflect on the role of different groups and networks in the policy process?
9. Can you please reflect on the values and beliefs of key or influential individuals?
10. Can you please reflect how the issue of obesity is framed (e.g., personal responsibility or environmental/ governmental responsibility), and how this played a role, if at all?

Other general reflections:

11. What do you think policy actors (i.e., advocacy groups, community organisations, local government) can learn to influence policy in support of obesity prevention?
12. To assist with my data collection can you please:
    a. Suggest any other individuals I should talk with to get insight into this policy development process?
    b. Suggest appropriate documents that would be useful for me to include into my dataset?

SUPPLEMENTARY FILE TWO: EXAMPLE QUALITATIVE DATA CODING CHART USED TO DEVELOP CAUSAL LOOP DIAGRAMS

Microstructure	Causal variable	Outcome variable	Relationship type/ Polarity	Text data showing causal linkage	Triangulation	
					Data	Method
1	Evidence of policy effectiveness	Support for policy from decision-makers	+	"New South Wales as a stakeholder were really, really beneficial in feeding us insights and evidence and I mean their evaluation actually that evaluation was key, I think, to having it through [the bill accepted through parliament]. Because all of the evidence that was published around the world was quite variable. Just having that evaluation [from a] similar context, and it was a very, very good evaluation." (DHHS Senior Policy Officer 9)<br><br>"…You want to impose a burden on businesses, [so] having a strong evidence base to demonstrate the benefits would balance the burden with the benefits of the proposal" (DHHS Food Safety Officer 1)<br><br>"One of the facts is that these sorts of laws do impose costs on businesses, and we never do that lightly, so you want to know that it is going to have an effect. The fact that there was a significant decrease in the kilojoules purchased as a result of the evaluation in New South Wales has been important evidence."- Ms Wooldridge (LPA), Member for Eastern Metropolitan<br>(Victorian Parliament Hansard, Legislative Council, 9 February 2017)<br><br>"I understand that in New South Wales, where this scheme has been operating, they have found that the average number of kilojoules consumed per meal has decreased by 15 per cent. That is a great result and something we look forward to having happen here in Victoria." - Ms Graley, (ALP), Member for Narre Warren South, Victorian Parliament Hansard, Legislative Assembly, Wednesday 12 October 2016)	Y	Y
